# Comprehensive Resource Utilization of Waste Using the Black Soldier Fly (*Hermetia illucens* (L.)) (Diptera: Stratiomyidae)

**DOI:** 10.3390/ani9060349

**Published:** 2019-06-13

**Authors:** Cuncheng Liu, Cunwen Wang, Huaiying Yao

**Affiliations:** 1 Ecology and Biological Engineering, School of Environmental Wuhan Institute of Technology, Wuhan 430073, China; liucc5735@126.com; 2 Research Center for Environmental Ecology and Engineering, School of Environmental Ecology and Biological Engineering, Wuhan Institute of Technology, Wuhan 430073, China; 3 Key Laboratory of Green Chemical Process of Ministry of Education, Wuhan Institute of Technology, Wuhan 430073, China; 4 Key Laboratory of Novel Reactor and Green Chemical Technology of Hubei Province, Wuhan Institute of Technology, Wuhan 430073, China

**Keywords:** black soldier fly (*Hermetia illucens* (L.)), organic waste, bioconversion, insect protein, insect grease

## Abstract

**Simple Summary:**

The black soldier fly *Hermetia illucens* (L.) (Diptera: Stratiomydiae) is an important resource and environmental protection insect with a large biomass, a high food conversion efficiency, and a high reproductive rate. *H. illucens* can be applied in environmental ecology and as an effective treatment for organic waste. Due to its broad feeding range, many types of organic waste (such as kitchen waste, livestock excrement, deteriorated fruits and vegetables, crop waste, and food-processing waste) can be converted into proteins, lipids, peptides, amino acids, chitin, vitamins, and polypides. The proteins and amino acids have been used to produce aquaculture feed and feedstuffs with high digestibility. In addition, the grease from *H. illucens* digestion has been successfully used as a raw material for biodiesel with good performance. Furthermore, the antimicrobial peptides and chitin extracted from *H. illucens* have high medicinal value. For the full development and utilization of this resource, studies of the biological characteristics of the *H. illucens*, its resource utilization of proteins and grease, and its environmental applications are crucial.

**Abstract:**

The black soldier fly, *Hermetia illucens* (L.) (Diptera: Stratiomyidae), is a saprophytic insect that can digest organic wastes, such as animal manure, plant residues, and food and agricultural wastes. In the degradation process, organic wastes are converted into protein, grease, and polypeptides, which can be applied in medicine, the refining of chemicals, and the manufacturing of feedstuffs. After their conversion by the *H. illucens*, organic wastes not only become useful but also environmentally friendly. To date, the *H. illucens* has been widely used to treat food waste and to render manure harmless. The protein and grease obtained via this insect have been successfully used to produce livestock feed and biodiesel. In this article, the biological characteristics, resource utilization of protein and grease, and environmental functions of the *H. illucens* are summarized. This article provides a theoretical basis for investigating potential applications of the *H. illucens*.

## 1. Introduction

*H. illucens*, was thought to originate from the South American savannah. It is widely distributed in temperate, subtropical, and tropical regions [1]. However, the recent discovery of a larva of *H. illucens* larva in the sarcophagus of Isabella d’Aragona (1470–1524) shows how the geographical origin and spread of this insect still represent an intriguing topic [2]. The *H. illucens* inhibits multiplication of the housefly [3], but unlike the housefly it does not invade human living environments, pollute the environment, spread diseases, or harm crops [4,5]. Research has identified the larvae of this insect as a type of saprophytic insect that feeds on industrial, agricultural, and household organic waste or plant and animal remains [6]. The larvae can digest harmful and pernicious bacteria, such as *Salmonella* spp. and *Escherichia coli*, in the process of organic waste decay [7,8,9], thereby reducing the harmful effect of such waste on the environment [7]. In early research, Sheppard and Tomberlin discussed the factors that influence *H. illucens* oviposition, which provided technical support for the artificial breeding of *H. illucens* [10,11] and subsequent development of the resource value of the *H. illucens*. Due to its wide dietary range, the *H. illucens* has been widely used to treat and render harmless food waste, manure [8], and diseased or dead livestock. *H. illucens* has been studied and utilized in many fields to favorable effect. A complete list of substrates that have been tested as feeding substrates for *H. illucens* larvae and the corresponding outcomes are provided in the table. It can be seen from the Table 1 that the effects on the development of the *H. illucens* vary among substrates. In additional to substrate type, environmental factors are key factors affecting the development of the *H. illucens*, contributing to variation among studies. In general, substrates rich in nutrients (protein and oil), are more suitable for the development of the *H. illucens*.

Organic wastes are converted by biotransformation into organic matter comprising polypeptide-containing proteins, lipids, peptides, amino acids, chitin, and vitamins. Therefore, *H. illucens* is regarded as a non-pest and beneficial insect. Insects are regarded as an important source of protein for the 21st century [26]. The digestibility of insect-derived protein can reach more than 70%, which approaches the digestibility of fish and meat protein and is significantly higher than that of vegetable protein. In addition, much progress has been made in research on the replacement of fodder with *H. illucens* protein [27,28,29]. Several studies have indicated that *H. illucens* can be induced to produce antimicrobial peptides, that have activity against many bacteria [30,31,32,33], and that chitin can be extracted from *H. illucens* puparium to produce chitosan [34]. Furthermore, *H. illucens* grease has substantial future potential in the development of high-tech products, such as biodiesel. Because of its abundant nutritional value, *H. illucens* offers considerable potential for exploitation. However, breeding on a scale appropriate for modern industrialization remains under-researched. Although studies have been conducted on, the application of chitin in the field of biomedicine; the industrial application of grease; and the efficient extraction of proteins, amino acids, peptides, and grease for the development of later high value-added products; detailed research on the feeding of *H. illucens* using agricultural waste as the primary raw material has not been reported. In this article, the development value and resource utilization of *H. illucens* and the use of this insect to treat and render harmless manure, agricultural wastes, and domestic wastes are summarized. This article provides a theoretical basis for research on the future ecological treatment of organic waste and other potential uses for *H. illucens*.

## 2. Biological Morphology

### 2.1. Morphological and Biological Characteristics

The *H. illucens* was first recorded by Linnaeus in 1738 and is mostly distributed between approximately 45° N and 40° S in the Americas [35]. *H. illucens* has been recorded in Beijing, Tianjin, Henan, Hebei, Shandong, Fujian, Sichuan, Yunnan, Hubei, Hunan, Guangdong, Guangxi, Hainan, Taiwan, Hong Kong, and other provinces or cities in China [36]. *H. illucens* possesses four distinct developmental stages: egg, larva, pupa, and adult. In nature, *H. illucens* lays its eggs in dry areas near wet, rotting organisms. Temperature, humidity, and light intensity are the main environmental factors affecting adult mating and egg laying [10]. The incubation time of the eggs is typically 4–14 days and varies with season, region, and temperature [37]. According to Kim, there are six instars in the larval phase [38,39], and the larvae range in size from 1.8 mm to 20 mm, with 20-mm larvae being referred to as mature larvae. *H. illucens* initiate feeding immediately upon hatching, with consumption rates increasing greatly after the 3rd instar. When the larvae reach the 6th instar, they cease feeding and initiate pupation, subsequently becoming adults. Adult females initiate egg laying approximately 2–3 days after mating, and female fecundity can reach 900 eggs/female [37]. The performance and nutrition of the *H. illucens* vary with substrate, and substrates rich in protein and oil are more conducive to the accumulation of protein and grease in this insect [40,41,42,43].

### 2.2. Ecological Characteristics and Environmental Stressors of Hermetia illucens

Natural ecological factors, such as parasites, microorganisms, hormone analogues, heavy metals, acid–base compounds, corrosive substances, and salt, affect the growth and development of insects [44,45,46,47]. Heavy metals in organic waste accumulate in *H. illucens*, which can affect the developmental period of this insect, increase larval mortality, and reduce the rates of pupation and emergence [48,49,50,51]. Studies indicate that different concentrations of Zn^2+^ have different effects on the developmental period of larvae, which mainly involve changes in weight, enzyme activity, total amounts of small molecule proteins and hemolymph levels of hormones in larvae [52,53]. Similarly, the weight, enzyme activities, levels of hormones, and, in particular, the contents of total sugars, proteins, and grease of larvae are affected by Cu^2+^. In addition, as Cu^2+^ accumulation in the larvae appears with high levels of the Cu^2+^ stress [54], the tolerance characteristics of mature larvae and pre-pupae can be affected by stress from alcohol toxicity, oxygen, and high osmotic pressure. Mature larvae and pre-pupae have been found to exhibit good resistance to these stresses, and their average mortality rates were found to decrease when the concentration of each stress factors (i.e., ethanol, mineral oil, and sodium chloride) is lower than 20–60%. In particular, at high levels of alcohol and osmotic pressure and low concentrations of oxygen, the development of larvae is threatened [55]. In addition, research has revealed that the Cd and Cr contents in the pupal shell are significantly higher than those in the pupa. This distribution pattern may be informative for studying the migration process of heavy metals in *H. illucens* [56]. Insect growth and development are also affected by temperature, light, and humidity. Certain appropriates temperature (27.5–37.5 °C) [57,58], light levels [59,60] and humidity (70%) [61] are more conducive to spawning, emergence, and conversion of organic waste in this insect. Several studies have found that hydrolytic proteins and organophosphates from *Bacillus subtilis* are present in the gut and skin of *H. illucens* larvae [62,63]. *Bacillus* has been reported to be a common microorganism that plays an important role in the intestinal tract of insects [64]. For example, a *Bacillus* strain exists in the gut of *Apis cerana japonica* (Japanese honeybee) that can inhibit *Paenibacillus*, which in the American honeybee can cause larvae to emit an unpleasant odor [65]. Bradley observed a *Trichopria sp* (Hymenoptera: Hamiidae) that can parasitize and lay eggs on the pupae of *H. illucens*, which likely affects development in *H. illucens* [66]. Although, the gut microbial communities (fungal and bacterial) in *H. illucens* larvae varies with substrate [63,67,68], identified communities in the gut of *H. illucens* have been found to be unique relative to those of other insects [69]. These unique communities, by possessing certain metabolic properties might be key in the conversion and digestion of complex organic waste by the *H. illucens* [70,71,72,73]. Thus, the gut microbiome in the larvae may play an important role in the conversion of organic waste.

### 2.3. Research on Artificial Breeding on Hermetia illucens

According to Tomberlin and Sheppard, the number of matings is proportional to the intensity of illumination in the range of 400–700 nm, and when the intensity of illumination is greater than 200 μ mol m^−2^s^−1^, the mating rate reaches 75%. However, at less than 63 μ mol m^−2^s^−1^, no mating behavior was observed [10]. In addition, the number of matings gradually decreases with time between 8:00–17:00, particularly before 15:00. Tomberlin and Sheppard achieved the highest mating rate with irradiation with an iodine tungsten lamp at a power of 500 W and an illumination intensity of 135 μ mol m^−2^s^−1^ under a temperature of 22 °C and a relative humidity of 60–70% The adult longevity (females: approximately 8–9 days; males: approximately 6–7 days) and hatching period of *H. illucens* are affected by morphological, biological, ecological factors, and environmental stress. Therefore, the density of *H. illucens* in the wild is low. In the 1970s, to promote resource utilization and large-scale production of *H. illucens*, Sheppard and Tomberlin started systematic research on the artificial breeding of *H. illucens*. They determined the ecological factors that affect mating and spawning and identified and summarized the that affect the survival rate of larvae and the development of the pupa prophase [10,17]. There were no significant effects of artificial feeding on the development and survival of pre-pupal *H. illucens*. However, the number of adults bred under fodder feeding was significantly lower than under natural feeding, and there were differences in the proportions of males and females. Therefore, to increase mating rates under artificial feeding, it is necessary to achieve the proportions observed in nature [74]. When peanut bran was used to feed the larvae, the effective conversion rate was 28.9%, this information was valuable for the standardization, scale expansion, and industrialization of the artificial breeding of *H. illucens* [75]. With the successful development of scientific, large-scale breeding of the *H. illucens*, the production of insect protein and oil has become possible [76,77]. Furthermore, organic wastes have been recycled and treated in an environmentally friendly way [78,79].

## 3. Resource Value of *Hermetia illucens*

Insects possess economic, ecological, and scientific value because their bodies (e.g., as food, medicine, or ornaments), products (e.g., secretions, excrement), behaviors (e.g., pollination, parasitism, preying on other organisms), cells and intracellular activities, and structures and functions (e.g., bionomics) can be used directly or indirectly by humans as resources. *H. illucens* is a resource insect with respect to its proteins, fats, and ability to convert organic waste. Current, research on *H. illucens* is primarily focused on its resource value and ecological value. The salivary glands and intestines of *H. illucens* can secrete digestive enzymes, and their digestive activities are significantly higher those of the enzymes secreted by houseflies [74,80]. Among these digestive enzymes, trypsin plays decisive roles in the digestion and transformation of organic wastes [81]. The gut microbiome in the larvae plays an important role in the conversion of organic waste [69,82]. The gut microbial communities (bacterial and fungal communities) in *H. illucens* are diverse and abundant, and they vary significantly with substrate [83], *Bacteroidetes*, *Proteobacteria*, *Firmicutes*, *Fusobacteria*, and *Actinobacteria* are the predominant bacterial taxa [63,68], and *Pichia*, *Geotrichum*, and *Trichosporon* are the predominant fungal taxa [67]. The functional and biological characteristics of *H. illucens* vary with variation in the compositions of the gut microbial communities [84]. These characteristics include oviposition, hatchability, longevity, conversion, and utilization efficiency of organic waste, and nutritional value [85,86].

**Figure 1:**
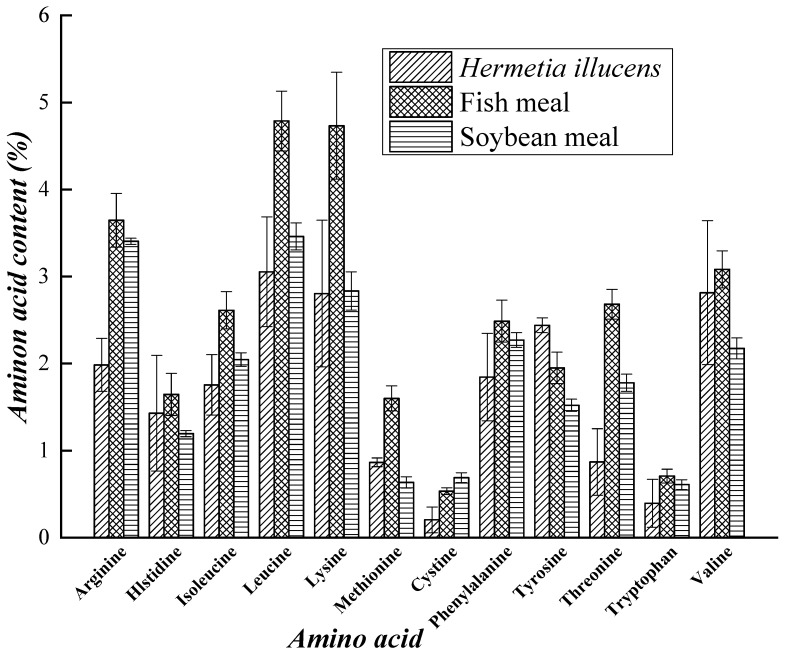
Amino acid contents of dried larvae, soybean meal and fish meal.

**Figure 2:**
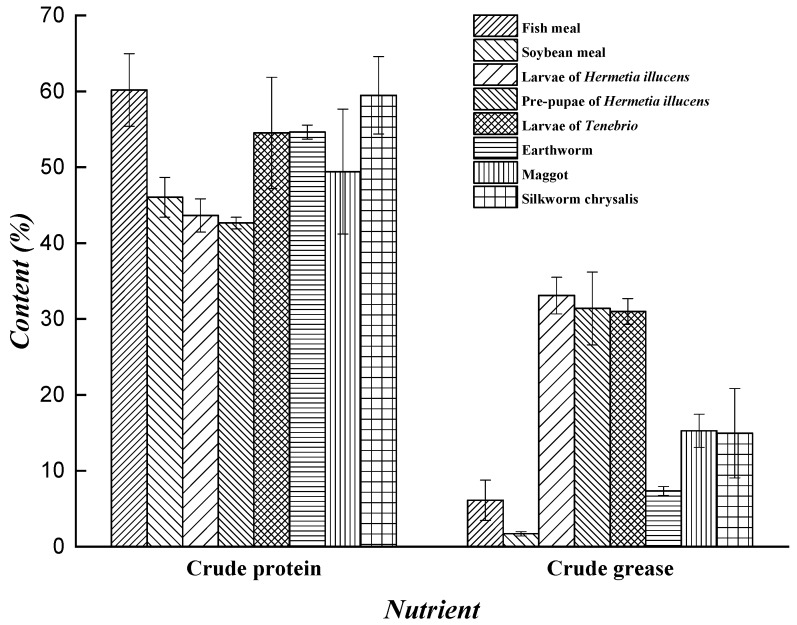
Nutrient contents of different insects, soybean meal and fish meal.

Due to the abundance of enzymes and microbes in the intestine of *H. illucens*, the larvae have a wider range of potential sources of food than do other saprophytic insects. The larvae can effectively digest and convert organic matter; therefore, high-quality fertilizers and organic matter can be acquired. Organic matter contains 20–70% protein, 30–60% amino acids, 10–50% fat, and 2–10% sugar. It also contains fatty acids, minerals elements, vitamins and other active substances that possess health functions for humans [87]. The nutritional value of *H. illucens* has been studied in the fields of industry, medicine, health care, and feed processing and has strong development potential. The protein derived from larvae has been used as animal feed [88,89] and as a substitute for soy flour or fish meal in formula feed for chickens, pigs, and fish [90]. The grease of the larvae has been used as raw material in the extraction of biodiesel [91,92]. Furthermore, *H. illucens* can be used as a raw material for the extraction of antimicrobial peptides [31,32,33]. The amino acid content of dried larvae, soybean meal, and fish meal [93,94], and the nutrient contents of different insects, soybean meal, and fish meal [95,96,97,98,99] are shown in Figure 1 and Figure 2 respectively.

### 3.1. Resource Value of *Hermetia illucens* Proteins and Minerals

The larvae of *H. illucens* are rich in amino acids and minerals. The nutritional value of larvae varies according to the type of organic waste the larvae are fed [96]. The *H. illucens* has been reported, as a good source of proteins and minerals [19,100]. Essential amino acids are abundant in the protein obtained from black soldier fly larvae. According to Sheppard and Newton, the leucine, isoleucine and valine contents of pre-pupa powder are much higher than those of fish meal, and the contents of all other amino acids except tryptophan are higher in pre-pupa powder than in soybean meal (Figure 1) [93]. Regarding mineral contents, the Fe, Mn, and Al contents of pre-pupa powder are much higher those in fish meal and soybean meal. The contents of some other minerals (Na, Mg, Zn) are also higher in pre-pupa powder than in fish meal and soybean meal, but those of K and Cu are slightly lower [93]. Compared with other insects, soybean meal and fish meal, the crude protein contents of the larvae and pre-pupae of *H. illucens* are slightly lower.

With the rapid development of stock breeding, an urgent need for protein feed has emerged. The protein from *H. illucens* can be used as feed or an additive for livestock and in aquaculture [89,101,102]. *H. illucens* has been validated as a feed additive or alternative for livestock and in aquaculture, and there has been much research on this application [103,104]. April Hari Wardhana has successfully harvested *H. illucens* as a protein substitute for animal feed [105]. Based on an analysis of the growth performance, serum indicators, and nutrient digestibility of pigs, Zhang et al. reported that larvae powder can be added as a protein feed or can replace fish meal and soybean meal in pig feed. When the larvae were used in broiler chicks feed, body weight gain was improved, and frequency of CD4+ T lymphocytes, serum lysozyme activity, and spleen lymphocyte proliferation were increased. These findings indicted that as feed, the larvae have prophylactic properties, stimulating non-specific immune responses and reducing the burden of *S. gallinarum* [106]. The nutritional value of the protein is greatly affected by the drying method, Huang et al. reported that conventional drying (60 °C) yielded a higher digestible indispensable amino acid score and better digestibility than did microwave drying [107]. It is necessary to identify improved methods to obtain protein and ensure its nutritional value and digestibility.

### 3.2. Resource Value of *Hermetia illucens* Grease

Although the grease content in *H. illucens* larvae is greatly affected by the rearing substrate [19,108,109,110], crude grease content is much higher in these larvae than in other insects, soybean meal, and fish meal (Figure 2) [95,96,97,98,99].

The resource value of the grease of *H. illucens* larvae has been demonstrated [18,111]. A total of 1200 *H. illucens* larvae can digest 1248 g of fresh cow dung by bioconversion in 21 days, and 15.8 g of biodiesel can be extracted from the grease of such larvae. When larvae were used to convert 1 kg of chicken manure, 1 kg of pig manure and 1 kg of cow dung, the amount of crude grease extracted by petroleum ether was 95.5 g, 60.4 g, and 38.2 g, respectively, which accounted for 30.1%, 29.1%, and 29.9% of the total biomass of the *H. illucens* larvae, respectively. The physicochemical properties of the crude grease of larvae are as follows: lodine value (the amount of unsaturation in fatty acids), 96 ± 2.4 gL/100 g; acid value, 8.7 ± 0.4 mg KOH/g; saponification value, 157.5 ± 6.2 mg KOH/g; melt point, 5 ± 0.3 °C; and peroxide value, 0.03 ± 0.01 meq/kg grease [112]. The physical and chemical fuel properties of biodiesel largely depend on the fatty acid distribution of the triglycerides used in the production. The fatty acid composition of larvae fed cow dung is enriched in myristic acid, palmitoleic acid, palmitic acid, oleinic acid, linolenic acid, stearic acid, and other acids [112]. Compared with biodiesel converted from rapeseed oil [112] and with reference to the EU biodiesel standard (EN14214), the parameters of the biodiesel converted from the crude grease of larvae fed on chicken manure, cow dung, pig manure, dairy manure, and restaurant waste met the standard and exhibited higher oxidation resistance [15,18,111]. Seven days after feeding 1000 larvae 1 kg kitchen waste, 64.9 g of larvae dry matter was obtained. After subsequent refining, 23.6 g of biodiesel with an ester content of 96.9% was obtained, and the fuel performance of this biodiesel almost met the EU biodiesel standard [113]. Experimental studies have indicated that *H. illucens* can effectively convert kitchen waste and rice straw into organic matter containing polypeptides by co-biotransformation and that the resulting crude grease can be refined into biodiesel that meets the EU biodiesel standard [16]. The grease from larvae has been used to replace traditional grease sources in feed formulations without adverse effects on animal performance and product quality [94,114,115]. When the grease of this insect was used to substitute soybean oil in the diets for broiler chickens, the productive performance, carcass traits, and overall meat quality were found to be satisfactory [116,117]. Similarly, when grease from *H. illucens* was provided as dietary grease to juvenile Jian carp, satisfactory results were obtained except for a decrease in lipid deposition in the intraperitoneal fat tissue of Jian carp [118].

Compared with typical energy-resource plants, *H. illucens* has advantages of high fertility and a short life cycle. In addition, *H. illucens* can convert livestock excrement and domestic waste into clean energy, which results in the resource utilization of organic waste. In addition, glucose and xylose have been shown to contribute to the accumulation of lipids in larvae, yielding a lipid content of 34.60% [119]. These findings indicate that, lignocellulose is a useful substance for lipid accumulation in larvae and that the *H. illucens* is a very promising organism for the conversion of lignocellulose [120,121]. Therefore, the conversion of organic waste into grease by *H. illucens* has substantial potential and research value.

### 3.3. Other *Hermetia illucens* Resource Values

The larvae and pre-pupae can be developed not only as sources of insect protein and grease but also as high-value resource materials for the extraction of antimicrobial peptides, chitin, and chitosan [122]. Osama Elhag et al. reported seven new gene fragments of three types of antimicrobial peptides obtained from *H. illucens* [123]. The induction and extraction of antimicrobial peptides from *H. illucens* revealed that the best period for induction and production was the 5th instar. In one study, the abdomens of 5th instar *H. illucens* larvae were punctured with needles dipped in *Escherichia coli* solution (3 × 10^12^ individuals/mL) and then soaked in *Escherichia coli* solution for 60 s. After larval feeding for 24 h the activity of antimicrobial peptides against *Escherichia coli* was better than that induced by ultrasound of 100 W for 20 min [124]. The hemolymph of larvae can be induced to produce antimicrobial peptide DPL4 [30,31], which exhibits antibacterial activity against Gram-positive bacteria, including *Staphylococcus aureus*, and the immunity of the larvae can be promoted. The active antimicrobial peptide induced by methanol solution has strong inhibitory effects on many bacteria. The crude antimicrobial peptide can inhibit *Staphylococcus aureus* well after purification, as evidenced from high-performance liquid chromatography. Compared with natural conditions, needle stimulation has been shown to result in stronger antimicrobial activity and a broader antibacterial spectrum of the antimicrobial peptide [30,31]. In addition, the antimicrobial peptide derived from *H. illucens* larvae has been shown to possess greater thermal stability than antimicrobial peptides derived from other insects and to exhibit good performance after repeated freezing and thawing. Storage time can reach 96 h at room temperature, and the suitable pH range is 5–9, which indicates good biological stability [125].

Chitin derived from crustaceans is a linear biological biopolymer [126] and has been widely used in the textiles industry, papermaking, agriculture and forestry, food, medicine, environmental protection, bio-engineering, and other fields [127,128]. The resource value of insects derives not only from their protein, grease, and antimicrobial peptides but also from their chitin. In recent years, chitosan has been successfully extracted from the pupa shell of silkworms, maggots, and pine caterpillars [129]. White or yellowish chitin was extracted from the pupa shell of *H. illucens* and successfully transformed into chitosan by Xu Qiyun for the first time. The extraction rates of chitin and chitosan were 12.3–14.3% and 83.2–86.3%, respectively, and the degree of deacetylation of the chitosan was 82.8%. Waśko and Bulak reported the physicochemical properties of chitin extracted from larvae and imagoes of *H. illucens*. The surface morphologies of the two chitin types were separately analyzed by scanning electron microscopy, and the crystalline index values of the chitins extracted from the imagoes and larvae were 24.9% and 35%, respectively. Compared with the crystallinity indexes (CrI) of chitin from other insects, those of chitin extracted from *H. illucens* were much lower. Low-crystallinity chitin has desirable adsorption properties [130], and chitin with a low CrI index exhibits high heavy-metal-removal efficiency due to its low diffusion resistance [131]. The chitin extracted from *H. illucens* larvae has unique physicochemical traits, suggesting *H. illucens* as a new source of this biopolymer for biotechnological applications [34]. Therefore, the resource value of the antibacterial peptides and chitin of *H. illucens* may become a topic of further investigation in the biomedical field.

### 3.4. Ecological Value of the Treatment of Organic Waste by *Hermetia illucens*

The treatment of organic waste by *H. illucens* larvae is gaining attention [69,110,132,133,134,135]. In addition, other types of waste are being explored as potential substrates for the sustainable use of *H. illucens* larvae biomass as food for humans and other animals [43,136,137].

Previous research has revealed that the ecological characteristics of *H. illucens* are similar to those of houseflies and that the two species appear to compete with one another. *H. illucens* fly has been shown to inhibit the breeding of houseflies [3,93]. In 1997, Sheppard successfully developed the first system for excrement treatment using *H. illucens*. Subsequently, research on the treatment of livestock excrement and domestic waste has been ongoing. In 1997, Dr. Oliver of the United States Environmental Technology and Engineering Corporation invented a *H. illucens* larvae biotransformer for domestic use. The biotransformer has been successfully used to treat municipal solid waste. In 2007, Dr. Xincheng An of the Guangdong Institute of Entomology designed a bioconversion system for the efficient large-scale treatment of organic waste; the system achieved satisfactory reduction, decontamination, and recycling of waste. Kitchen waste, livestock excrement, deteriorated fruits and vegetables, crop waste, and food-processing waste can be quickly and effectively converted by *H. illucens* larvae because of this insect’s wide range of potential foods. The converted waste then forms the biomass of *H. illucens* larvae itself, such that *H. illucens* larvae can be applied in environmental ecology and as an effective treatment for organic waste [138,139].

Environmental safety assessment has shown that *H. illucens* consumes only small amounts of plant juice and does not carry human pathogens. Therefore, *H. illucens* has important ecological value, appears not to harm human beings or their environment, and poses no threat to crops.

#### 3.4.1. Research on the Treatment of Excrement by *Hermetia illucens*

The accumulation of livestock excrement that cannot be promptly and effectively treated leads to environmental pollution, primarily through air, water, and soil pollution [140,141]. At present, the methods of converting or utilizing livestock excrement are mainly restricted to excrement use in high-temperature compost, as an energy source, and in feedstuff production [142]. In the late 1970s, *H. illucens* larvae began to replace houseflies in the bioconversion of livestock excrement, and substantial progress rapidly followed [94,143]. Recently, a model for converting livestock excrement by *H. illucens* larvae has been piloted and promoted for sustainable agricultural production in the United States. In 1994, Professor Sheppard of the University of Georgia (USA) established an efficient, low-cost a manure treatment system using *H. illucens* larvae. The system converted half the manure into protein (42%) and grease (35%) as evidenced by the characteristics of *H. illucens*, and the nitrogen content and the abundance of *E. coli* were significantly reduced [7,17]. Fermented and fresh pig manure was also converted by *H. illucens* larvae, with conversion rates of 23% and 28%, respectively. After conversion, the content of organic matter in the manure was 71.9%, the total nutrient content (N + P_2_O_5_ + K_2_O) was 8.76%, and the pH value was 7.3. These nutrient values fully met the standard for organic fertilizer [144]. With the amounts of fecal accumulation and metallic elements (except iron) being significantly reduced [145]. Cow dung, swine manure and chicken manure converted by *H. illucens* larvae can be used as high-quality organic fertilizer and produce no offensive smell. In particular, cow dung converted by this insect becomes less solid, and when as an organic fertilizer, can increase the growth of pasture. In addition, the nitrogen, phosphorus and potassium contents of pig manure were found to decrease by 55.1%, 44.1%, and 52.8%, respectively, following conversion, and organic matter formed. These observations indicate that *H. illucens* larvae offers significant economic and environmental benefits in the treatment and conversion of livestock excrement [111]. In addition, the treatment system has been adopted to the treatment of waste from animal farming and has achieved satisfactory results [146,147,148,149]. *H. illucens* larvae can effectively convert human feces from source separation toilets or fecal sludge from onsite sanitation systems [9,150,151]. Research has shown that after conversion, the pH of human feces increases, the total amounts of solid and total volatile solids and the abundance of *Salmonella* significantly decrease, and the level of total ammonia nitrogen improves. No significant effect of such treatment has been observed on enterococci, phage 174, or *Ascaris* eggs. These outcomes indicate the environmental and ecological value of such treatment [12].

#### 3.4.2. Research on the Treatment of Other Organic Waste by *Hermetia illucens*

In addition to excrement, kitchen waste, coffee grounds, palm seed meal, agricultural waste, and other organic waste can be effectively converted by *H. illucens* larvae [152]. Kitchen waste contains not only large amounts of organic components, such as starch and cellulose, but also fat, salt, and abundant trace elements. This form of organic waste continuously affects the environment. Another common form of organic waste is fruit and vegetable waste from food companies or markets. At present, treatment methods for organic waste primarily include incineration, landfills, fodder, anaerobic digestion, aerobic composting, and earthworm compost [153]. However, these treatment methods cannot satisfy the requirements of environmental protection and a high degree of resource utilization. *H. illucens* larvae has been applied to the conversion of kitchen, fruit and vegetable waste [13,20,146,147,154,155]. Research has shown that among the larval instars of *H. illucens*, 6th instar larvae exhibited the highest survival rate and the highest conversion rate in converting kitchen residue, particularly when the moisture level was 60%. Regarding the dry matter of old mature larvae, values of 44.7% protein and 37.2% grease were obtained [113]. In addition, the NH^4+^ of the kitchen waste was 5–6 times higher following treatment than before treatment; such conversion has the potential to offset the nitrogen accumulation caused by fertilization [156]. Therefore, there are substantial prospects for the conversion and treatment of kitchen, fruit and vegetable wastes by *H. illucens* larvae. More importantly, effective treatment and conversion of municipal organic solid waste and industrial organic waste by larvae has been achieved [43,154,157,158]. These advances provide a basis for the large-scale cultivation of *H. illucens* and the large-scale treatment of organic wastes [136]. In 2015, based on the effective conversion of organic wastes by *H. illucens* larvae, a specialized *H. illucens* larvae breeding base was established in Tieyong Town, Huidong County, Guangdong Province. In the same year, the output and the kitchen waste treatment capacity were 1.3 t/day and 7.8 t/day, respectively. The bioconversion of a mixture of rice straw and kitchen waste was investigated. A total of 2000 *H. illucens* larvae were used to convert 1.0 kg of an organic waste mixture (30% rice straw; 70% kitchen waste). After 10 days, 65.5% of the cellulose, 56.3% of the hemicellulose, 8.8% of the lignin, 91.6% of the protein, and 71.6% of the fat had been digested by *H. illucens* larvae and converted into their own biomass. Therefore, the transformation of agricultural waste using *H. illucens* larvae has excellent prospects.

#### 3.4.3. Ecological Value of *Hermetia illucens* for the Treatment of Organic Waste

After the conversion of organic waste by *H. illucens* larvae, organic waste accumulation is reduced, and pollution and harm to the environment are decreased. In its ecology, *H. illucens* fly is similar to the housefly. However, the larvae of *H. illucens* fly can produce pheromones that are repellent to houseflies, which results in no oviposition or breeding of houseflies in excrement. Similar effects of *H. illucens* fly against other noxious insects, such as *Tenebrio molitor*, have been observed [3,93]. Following the inoculation of chicken manure with labelled *E. coli* and *Salmonella enterobacter*, the use of *H. illucens* larvae to convert the manure was found to reduce the abundances of these pathogens by more than 2000 times [7]. The larvae not only inhibited *Escherichia coli*, *Staphylococcus aureus*, and *Salmonella* but also displayed the ability to escape the symbiotic matrix with *Salmonella*, especially at a temperature of 27 °C. In addition, the larvae affected the growth of *E. coli* in cow dung and showed good antibacterial properties. Furthermore, it has been shown that during conversion, some organic pollutants, such as antibiotics and pesticides, are degraded [159], the accumulation of metals in the organic waste is decreased [48,160,161,162,163], and the abundances of pathogens and harmful microorganisms are reduced [7,8,164,165] by the larvae. In addition, the conversion of organic waste by *H. illucens* larvae results in no accumulation of antibiotics, pesticides, dioxins, polychlorinated biphenyls or polyaromatic hydrocarbons in the insect body; only heavy metal accumulation has been observed [159,161,166,167]. Thus, in addition having saprophytic characteristics, *H. illucens* larvae is an important insect for environmental protection because it has the ability to digest or decompose harmful bacteria in the environment and reduce the harmful effects of waste on the environment.

## 4. Conclusions and Prospects

Due to its biological characteristics, the *H. illucens* has been widely used for the environmentally friendly treatment of common organic waste, such as excrement, kitchen waste, domestic waste, and agricultural waste. Importantly, such treatment not only converts the organic waste to useful and harmless forms but also results in the conversion of the nutriment to insect biomass. The proteins and grease of this insect have been applied to the breeding industry and the fine chemical engineering industry. In addition, the antimicrobial peptides and chitin obtained from *H. illucens* are important raw materials for biology, medicine, and food production. However, the biological characteristics and resource value of this insect have not been used effectively or sufficiently because of the low level of industrialization. Although this insect has been applied to the treatment of organic wastes and the feed industry, the industrialized, large-scale breeding of *H. illucens* remains in the exploratory stage. Reports on the detailed process and mechanism of conversion are lacking, although some research on the gut microbial communities of the larvae has been performed. Crop straw is an important source of biomass energy, but its utilization efficiency is low, and its processing is challenging due to its physicochemical properties. The bioconversion of agriculture waste by *H. illucens* is in very early stages, and studies of the mechanisms of the bioconversion and degradation of crop straw by *H. illucens* in cooperation with microorganisms are needed.

With the rapid development of intensive and modern industry, agriculture and livestock farming, problems related to food, the environment and energy will be aggravated. Research on the comprehensive resource utilization of waste using the *H. illucens* will receive increasing attention. Studies of the process and mechanism of conversion, including the interactions with microorganisms, are needed, as are studies of the acquisition and safety evaluation of nutrients from this insect. The results of such efforts might encourage the development of industrialized, large-scale breeding and a processing industry based on *H. illucens* as well as the safe and useful treatment of organic wastes. These developments will play an important role in agriculture, industry and human health.

## Figures and Tables

**Table 1:** Complete list of tested substrates as feed for *H. illucens* larvae (NA: not available).

Substrate	Conversion Rate of Substrate (%)	Output (%)	Survival Rate (%)	Larvae Composition (%)	References
Poultry feed	23–70	1.4–16	40–93	P: ~40, G: ~35	[12,13,14]
Kitchen waste	~60	NA	NA	P: ~40, G: ~35	[15,16]
Livestock waste	25–53	8–22	71–99	P: ~40, G: ~30	[17,18]
Domestic or municipal organic wastes	39–79	~14.5	NA	NA	[9,19,20]
Agro-industry by-products	6.3–27	4–6	NA	P: ~30, G: ~28	[21,22,23,24]
Crop straw	9–68	5–10	51–98	NA	[25]

Output refers to the ratio of the increase in the insect biomass to the substrate-induced biomass reduction (dry weight). P; Protein contents of larvae; G: Grease contents of larvae.
